# Acetaminophen study yields new insights into neurobiological underpinnings of empathy

**DOI:** 10.1152/jn.00723.2016

**Published:** 2016-10-05

**Authors:** John Tully, Marija M. Petrinovic

**Affiliations:** ^1^Department of Forensic and Neurodevelopmental Sciences, Institute of Psychiatry, Psychology and Neuroscience, King's College London, London, United Kingdom; and; ^2^Sackler Institute for Translational Neurodevelopment, Institute of Psychiatry, Psychology and Neuroscience, King's College London, London, United Kingdom

**Keywords:** acetaminophen, empathy, pain, paracetamol

## Abstract

Empathy is a cornerstone of social behavior, impairments of which are characteristic of neuropsychiatric disorders such as autism and psychopathy. According to the “shared representations” theory, empathy relies on neural processes similar to those underpinning the first-hand experience of a given emotion. A recent study by Mischkowski, Crocker, and Way (*Soc Cogn Affect Neurosci* 11: 1345–1353, 2016) provides novel insights into neurobiological underpinnings of empathy by demonstrating that acetaminophen, a widely used painkiller, reduces empathy for other's physical and social pain.

social relationships are cornerstones of human societies. Empathy, the ability to perceive, vicariously experience, and respond to the emotions of other people, is central to social relationships given it is thought to be a key motivating factor for prosocial behavior (Decety et al. 2016). In contrast, empathy has been shown to be impaired in a number of neuropsychiatric disorders, including psychopathy and autism spectrum disorder (Decety et al. 2016). Therefore, understanding the neurobiological underpinnings of empathy holds promise in developing interventions to improve social interactions in individuals with these disorders.

Although empathy has been extensively discussed and investigated by philosophers and psychologists, only recently has it attracted the interest of neuroscientists (Preston and de Waal 2002). This was prompted mainly by the discovery of mirror neurons, a class of visuomotor neurons in brains of macaques that respond to both action execution and observation of the same action. On this basis, Preston and de Waal (2002) suggested a “shared representations” hypothesis, wherein empathy relies on neural processes similar to those underpinning the first-hand experience of a given emotion. In support of this, subsequent functional magnetic resonance imaging (fMRI) studies in humans have shown that vicariously experiencing pain activates (part of) the neural network that is also activated when we are in pain ourselves (Lamm et al. 2011). Specifically, observing others in pain activates anterior insula (AI) and the anterior cingulate cortex (ACC), brain regions that are also activated during one's own experience of pain (Lamm et al. 2011). However, overlap of neural activations does not necessarily mean that equivalent neural functions are engaged by empathy and the corresponding first-hand emotion experience. Indeed, in the field of fMRI research it is well known that the same brain structure can be activated by a variety of tasks and functions. A more causal demonstration that representation of another's emotion is specifically grounded in neural mechanisms that also serve the first-hand emotion experience was recently provided by Rutgen et al. (2015). In that study, Rutgen et al. demonstrated that experimentally reducing the first-hand experience of pain by means of placebo analgesia equivalently reduces empathy for pain and that this is accompanied by matching fMRI activation changes in brain areas associated with shared activations in pain and empathy for pain. Intriguingly, their findings also imply that analgesics may have the unwanted side effect of reducing our concern for others' pain and suffering.

Important support for the latter was recently provided by the study of Mischkowski et al. (2016), who showed that acetaminophen (also known as paracetamol), one of the world's most widely used nonprescription analgesics, reduces empathy for pain in others. In the United States alone, 23% of the population (including children) use acetaminophen-containing drugs each week to relieve pain and/or fever (Mischkowski et al. 2016). Until recently, the side effects of acetaminophen were considered to be well-known (Graham et al. 2013). However, recent studies have uncovered some previously unrecognized psychological side effects related to acetaminophen's use, ranging from diminished existential anxiety and reduced evaluation and response to both negative and positive stimuli to reduction in psychological pain caused by social rejection (see Mischkowski et al. 2016).

On the basis of observations that self-experienced pain and empathy for pain in others involve (partially) overlapping neural circuits (Lamm et al. 2011) and that placebo analgesia reduces vicarious experiences of others' pain (Rutgen et al. 2015), Mischkowski et al. (2016) hypothesized that *1*) using a painkiller to pharmacologically inhibit neural circuits for experiencing one's own pain should also impair empathy for another's pain and that *2*) in keeping with the “shared representations” theory, acetaminophen should also impair empathy when witnessing another person in social pain because its consummation reduces the first-hand pain of social rejection (DeWall et al. 2010).

The authors tested these hypotheses in an elegantly designed double-blind, placebo-controlled study on healthy college students. The study participants received either 1,000 mg of acetaminophen or a placebo. While waiting for the drug to take effect, the participants were allowed to get acquainted with each other. Afterwards, all the participants were subjected to short, loud blasts of white noise (75–105 dB), after which they were asked to rate the “affective noise pain,” i.e., unpleasantness of the noise, for themselves. Their empathy assessment was based on two different types of ratings. One evaluated cognitive aspects of empathy (perceived noise pain, or how unpleasant they thought the noise would be for another study participant), and the other evaluated more emotional aspects (personal distress and empathic concern when witnessing others' pain). Compared with the placebo group, participants who received acetaminophen rated the noise blasts as being less unpleasant for themselves. They also thought that such noise would be less unpleasant for other participants and expressed less personal distress and empathic concern for others experiencing the same noise blasts. Hence, the authors demonstrated that by alleviating first-hand pain experience, acetaminophen affects not only how much pain we think others are experiencing but also how much of that pain we feel or vicariously experience ourselves. Thus, by using pharmacological modulation of self-experienced pain, Mischkowski et al. (2016) have provided more direct evidence for the “shared representations” theory of empathy (Preston and de Waal 2002). They also raised further concern about the possible psychological side effects of acetaminophen. Specifically, by diminishing our ability to “put ourselves in others' shoes,” acetaminophen may reduce willingness to help others in distress. The observed reduction in empathy is of further concern, because as a driver of prosocial behavior, empathy also involves inhibition of aggression (Decety et al. 2016). This is important at a societal level, where aggression and violence have enormous human and financial costs. The possibility of increased aggressive behavior related to acetaminophen use therefore warrants assessment in future studies.

Mischkowski et al. (2016) then went on to test their second hypothesis, that acetaminophen might also affect empathy for others' social pain. Each study participant was therefore asked to watch an online ball-tossing game (Cyberball) that purportedly involved three people they had just met, i.e., other study participants. In the game, two of the participants excluded the third one from the activity, thus providing a cue for the observing participant to empathize with someone going through a socially painful experience. Afterwards, participants were asked to rate the emotional pain of the socially ostracized participant. Those individuals who took acetaminophen rated the pain and hurt feelings of the socially rejected participant as being not as severe as did the participants who took the placebo. Acetaminophen-treated participants also showed less empathic concern and distress in response to others' social pain than did participants treated with placebo. Thus, by complementing the previous report of acetaminophen-diminished self-experienced pain due to social rejection (DeWaal et al. 2010), this finding of Mischkowski et al. (2016) further substantiated the “shared representations” theory of empathy.

Surprisingly, however, the bias-corrected bootstrap mediation analysis showed that the auditory pain experienced by the participants accounted for the effect of acetaminophen on reduced empathy for others' noise pain, but not for empathy for others' social pain. This finding is of particular importance because it implies that physical pain and pain of social rejection might not share common neural mechanisms as previously suggested by virtue of overlapping fMRI activity (Eisenberger et al. 2003). Interestingly, Woo et al. (2014) also recently challenged this notion by identifying distinct multivariate fMRI patterns unique to each type of pain, i.e., physical or social, that are colocalized at the gross anatomic level. Thus this finding of Mischkowski et al. (2016) adds further support for the emerging idea that AI and ACC, two brain regions implicated in processing of both physical and social pain, contain multiple, distinct neural population codes that encode distinct mental processes. Because acetaminophen has been shown to modulate the activity of those two brain regions (DeWaal et al. 2010), it then stands to reason that this drug might affect empathy for different types of pain. It should also be noted that the effect of acetaminophen on empathy for social pain demonstrated by Mischkowski et al. (2016) appeared to be smaller than the effect on empathy for physical pain. This was reinforced by two additional experiments in the same study, in which the acetaminophen-treated participants reported less empathy toward social than physical pain of fictional characters.

A further consideration relevant to this study is that empathy is thought to broadly consist of both cognitive empathy (knowing what others are feeling) and affective empathy (the emotional response to what others are feeling). A recent meta-analysis by Fan et al. (2011) suggested that cognitive and affective empathy can also be distinguished at the level of regional activation patterns. Within the core network of empathy, independent of task and stimulus type, affective empathy was more likely to activate the right AI and cognitive empathy was more likely to activate dorsal medial cingulate cortex, whereas the left AI was involved in both types of empathy (Fan et al. 2016). In light of these findings, it is noteworthy that Mischkowski et al. (2016) reported a moderately strong effect of acetaminophen on affective empathy, whereas its effect on cognitive empathy was rather small. This observation is suggestive of differential regulation of the underlying neural circuits by acetaminophen and thus encourages its further use in “proof-of-concept” studies aimed at uncovering the neurobiological underpinnings of empathy.

How a single dose of acetaminophen causes reduction in empathy is unclear. The analgesic effects of acetaminophen can be reduced by inhibitors of many endogenous neurotransmitter systems, including serotonergic, cannabinoid, and opioid systems (Graham et al. 2013). The latter was recently shown to underlie the empathy-reducing effects of placebo analgesia (Rutgen et al. 2015), thus suggesting blockade of opioids as a possible starting point in the quest for mediators of acetaminophen's psychosocial effects. Previous research on the neurochemical basis of empathy has mostly focused on oxytocin, a neuropeptide that modulates emotion recognition accuracy as well as trust, generosity, and cooperation in humans (Decety et al. 2016). Tentative evidence that both oxytocin and acetaminophen affect empathy for other's pain by modulating activity in the AI and ACC (Bos et al. 2015) raises the intriguing possibility that both may exert their effects on interacting, and likely counteracting, neurochemical systems.

As we have highlighted, given the widespread use of acetaminophen, the suggestion that it reduces empathy might provoke cause for concern due to potential societal effects on empathy and aggression. Indeed, the study of Mischkowski et al. (2016) has received considerable attention in the media. However, as in many other instances when neuroscience has entered the public sphere, much of this coverage was somewhat simplistic, even sensationalistic, not providing audiences with sufficient detail to enable measured judgement on the quality and meaning of the research findings. Although more research on the social side effects of this medication is warranted, we want to draw attention to the fact that this study was carried out in healthy participants who were not experiencing actual physical pain before taking acetaminophen. Therefore, it is not clear if the link between taking acetaminophen and lower empathy levels would also be present in people taking the drug to deal with the real physical pain. Moreover, it has been shown previously that our physical state is an important factor that affects our propensity to behave empathically. Generally, personal experience of pain or social stress makes individuals less likely to empathize with others (Decety et al. 2016). Accordingly, alleviating one's own physical pain by using a painkiller is more likely to increase the empathy for the pain and suffering of others. In keeping with this, acetaminophen treatment of pained nursing home residents was shown to increase their social engagement (Chibnall et al. 2005). On balance, it is likely that any empathy reduction caused by acetaminophen would be outweighed by these and other benefits of its analgesic effects.

To further our understanding, we suggest that acetaminophen's effects on empathy be compared in healthy, pain-free participants vs. those in physical pain (either chronic or acute). In addition to self-reports, such a study would ideally involve fMRI measurements of neural activity to link behavioral/psychological representations with their neural underpinnings. Furthermore, it should be recognized in future research that like most neurocognitive and affective domains, empathy in individuals is not a binary entity but lies on a spectrum with considerable normal variation. Acetaminophen, although termed an “empathy killer” (Mischkowski et al. 2016), was more accurately shown to be a modest modulator. This being said, acetaminophen might indeed be a valuable pharmacological tool in “proof-of-principle” studies to tease apart neurobiological underpinnings of empathy. In turn, those studies could also help us to gain more precise knowledge of its analgesic mode(s) of action.

In summary, Mischkowski et al. (2016) have unveiled previously unrecognized psychosocial side effects of a popular over-the-counter painkiller, which may render us less appreciative of the pain and suffering of others. However, because the current study was performed on healthy, pain-free individuals, it is not clear if the link between taking the drug and lower empathy levels would also be present in people taking acetaminophen to ease their physical pain. Thus more research will be required to better understand the apparently complicated relationship between painkillers and empathy, but the endeavor is certainly warranted. Gaining new insights into the neurobiological underpinnings of empathy holds promise in enabling development of new pharmacological treatments for neuropsychiatric disorders characterized by social dysfunctions.

## GRANTS

This work was supported by a Wellcome Trust Clinical Research Training Fellowship (to J. Tully).

## DISCLOSURES

No conflicts of interest, financial or otherwise, are declared by the authors.

## AUTHOR CONTRIBUTIONS

M.M.P. conceived and designed research; J.T. and M.M.P. drafted manuscript; J.T. and M.M.P. edited and revised manuscript; J.T. and M.M.P. approved final version of manuscript.
